# A Computational Framework to Emulate the Human Perspective in Flow Cytometric Data Analysis

**DOI:** 10.1371/journal.pone.0035693

**Published:** 2012-05-01

**Authors:** Surajit Ray, Saumyadipta Pyne

**Affiliations:** 1 Department of Mathematics and Statistics, Boston University, Boston, Massachusetts, United States of America; 2 Department of Medical Oncology, Dana-Farber Cancer Institute, Harvard Medical School, Boston, Massachusetts, United States of America; 3 Broad Institute, Massachusetts Institute of Technology and Harvard University, Cambridge, Massachusetts, United States of America; University of Illinois at Urbana-Champaign, United States of America

## Abstract

**Background:**

In recent years, intense research efforts have focused on developing methods for automated flow cytometric data analysis. However, while designing such applications, little or no attention has been paid to the human perspective that is absolutely central to the manual gating process of identifying and characterizing cell populations. In particular, the assumption of many common techniques that cell populations could be modeled reliably with pre-specified distributions may not hold true in real-life samples, which can have populations of arbitrary shapes and considerable inter-sample variation.

**Results:**

To address this, we developed a new framework flowScape for emulating certain key aspects of the human perspective in analyzing flow data, which we implemented in multiple steps. First, flowScape begins with creating a mathematically rigorous map of the high-dimensional flow data landscape based on dense and sparse regions defined by relative concentrations of events around modes. In the second step, these modal clusters are connected with a global hierarchical structure. This representation allows flowScape to perform ridgeline analysis for both traversing the landscape and isolating cell populations at different levels of resolution. Finally, we extended manual gating with a new capacity for constructing templates that can identify target populations in terms of their relative parameters, as opposed to the more commonly used absolute or physical parameters. This allows flowScape to apply such templates in batch mode for detecting the corresponding populations in a flexible, sample-specific manner. We also demonstrated different applications of our framework to flow data analysis and show its superiority over other analytical methods.

**Conclusions:**

The human perspective, built on top of intuition and experience, is a very important component of flow cytometric data analysis. By emulating some of its approaches and extending these with automation and rigor, flowScape provides a flexible and robust framework for computational cytomics.

## Introduction

Flow cytometry is one of the most commonly used platforms in clinical and research labs worldwide. It is used to identify and characterize types and functions of cell populations in a sample by measuring the expression of specific proteins on the surface and within each cell. In recent years, intense research efforts have focused on automated analysis of flow cytometric data, especially for cell population identification [1]–[7] and flow data preprocessing [8]–[11].

Flow cytometric data consists of per cell measurements (or *events*) in the form of scattering of light and fluorescence intensity of fluorophore-conjugated markers. In a typical flow data analysis workflow, a human analyst visually inspects 2-dimensional scatterplots of a sample, where the dimensions could be scatters, marker intensities, or a combination of these, and she demarcates (or “gates”) specific populations of interest such as live cells, lymphocytes, etc. Often gates are drawn around visually discernible congregations of events. For instance, for live gating, the dead cells or debris could be discerned by their low cell size and granularity, which appear as a distribution of points with low forward- and side-scatter values. The manual approach to gating is, however, labor-intensive and subjective, and gating results can vary considerably from one analyst to another. For large-scale, reproducible flow data analysis, it is therefore necessary to design new algorithmic approaches for automated detection of cell populations.

While a variety of new algorithms have been proposed to automate gating, in general they have some important limitations. Often these algorithms use statistical clustering approaches that model cell populations as distribution of points which are assumed to have a certain pre-specified form, e.g. Gaussian kernels [12], [13]. However, assuming the large variety of cell populations characterized with flow cytometry to have one common, pre-specified shape, or the user to have a priori knowledge of the number of populations in a sample, as often required by such approaches, may neither be realistic nor necessarily lead to the best results. In addition to assuming the user to input model specifications that she may be unaware of in advance, ironically what she does know probably better than anyone else - her intuition into the data in hand - goes largely unutilized.

A serious drawback of most of the current automated gating methods is that they almost entirely ignore the key aspects of human perspective and intuition that guide the manual gating process. Clearly the task of gating relies on expert understanding of the underlying biology of the experiment - in terms of both design and outcome - as well as the different factors involved such as markers, dyes as well as the instrument under consideration. While machine learning techniques have traditionally been employed for understanding tasks that involve human faculties such as visual perception that guides the gating process, we believe a mathematically intuitive and syncretic approach may be better suited to address both of the above limitations and thereby improve automated gating. To emulate the subjective yet often quite reliable gating steps as executed by a trained human expert, an algorithmic framework must first be able to mathematically represent the flow data in terms of a “global” perspective, and then identify the more complex and inter-connected population structures therein. With the right mix of precision and intuitive flexibility, such a framework can best serve the needs of a number of problems in cytometric data analysis.

We present flowScape, a new computational framework to automate gating by emulating the human perspective. To achieve this, flowScape follows four steps: (a) mapping the data landscape with modal clusters, (b) building a hierarchical structure connecting the modal clusters, (c) performing ridgeline analysis to isolate the populations, and (d) constructing flexible, sample-specific templates to automate gating. Thus flowScape is designed to capture the best of two worlds: inferential properties of model based clustering and the flexibility of non-parametric techniques. Below we describe these steps in further detail. It begins with (a) a novel mapping of the multi-dimensional data landscape of a given flow cytometric sample, which creates a global overview of the data. However this overview is created with precision and rigor by characterizing regions in the landscape in terms of varying densities of points. These regions could be of arbitrary shapes but each of these are concentrated around a *mode* – resulting in modal clustering of the data. On top this, flowScape then (b) builds a hierarchy of the modal clusters to allow the user to perceive the inter-connected populations at multi-level resolutions. Such distribution could vary from rare, isolated events to the entire landscape at the topmost level. This representation offers unique advantages to flowScape. Inter-connected features in the landscape can now be characterized formally, and even separated objectively, using the hierarchical representation. In fact, viewing the data as a (high-dimensional) terrain, this is similar to traversing from one peak (or mode) to another across the intermediate slopes and valleys defined by changing altitude (or densities). This allows flowScape to (c) construct a ridgeline connecting the more complex population structures in the landscape. Since the ridgeline’s altitude at any point is proportional to the density of events at that location, it offers the objective means to isolate overlapping subpopulations from one another which may be otherwise difficult to achieve via automation. Often in manual gating, such a demarcation step is conducted with human intuition albeit in a subjective manner – flowScape combines flexibility with objectivity.

Notably, the modal clusters of flowScape are high-dimensional and unrestricted in shape. This offers flowScape a unique opportunity to improve the automation of the gating process. The modal clusters are used by flowScape to (d) construct dynamic, sample-specific templates for detecting populations not by their absolute coordinates but the corresponding congregation of events. Taking a semi-supervised approach, flowScape enables the user to construct templates of target populations in a training set of samples. Then those templates can be applied to new batches of samples to automatically identify the analogous features – in terms of their densities and not rigid locations – in a flexible, sample-specific manner. This capacity of flowScape generalizes gating and supports automated analysis of large cytometric cohorts. Similarly, flowScape may be useful for many common applications such as determining the optimal data transformation per flow channel, gating of live cells and lymphocytes, etc. For demonstration, we applied flowScape to multiple flow cytometric data sets, both published and newly generated, and also illustrated its advantages over other existing methods.

## Materials and Methods

We describe the methodology used in flowScape both as a general algorithm as well as in terms of particular applications in flow data analysis.

### Modal Clustering Methodology

Our formal approach to map the landscape in a multi-dimensional space of flow events utilizes two statistical concepts: a *mode* and a *density* function. Mathematically a mode is evaluated as the local maximum of the density function defined on the chosen data dimensions. Let a flow sample consist of *n* points 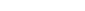 distributed in a multi-dimensional space measured either as scattering of light or fluorescence intensity (or “expression”) of markers per cell. A density function *f*(*x*) based on the data 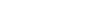 can be defined as in terms of relative frequency of points distributed around a specific mode. Traditionally, such distributions are perceived by a human analyst and characterized in terms of their concentration at a specific location rather than conforming to some pre-specified form or shape. In that direction, flowScape adopts the strategy of partitioning the event-space into modal clusters – in terms of regions of the data landscape where the density is relatively high, and surrounded by those where the density is relatively low. Yet often a complex landscape cannot be mapped optimally as merely a dichotomized collection of high peaks and low valleys, which therefore calls for a more robust algorithmic approach as described below.

In flowScape, we begin with the construction of the density of the data landscape for a given sample, and then determine the modes of that function. Although mode-counting or mode hunting has been extensively used as a clustering technique (see [14]–[19] and references therein), most implementation are limited to univariate data. Detection of modes in higher dimensions using standard optimization methods present a major technical and computational challenge [17], we used a Modal Expectation-Maximization (or “Modal EM”) approach [20], which allows us to address this problem with precision and efficiency. We begin by considering the kernel on every data point 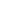 as individual mixture components of equal weight, and adapt and apply the EM [21] techniques developed in the parametric modeling paradigm. The modes of the kernel densities are calculated as distinct values from the set of values to which the Modal EM converges starting from each data point. Another obvious statistical challenge is the choice of the smoothing parameter for computing the density of the landscape. Based on the theory on generalized quadratic distance [22]–[24], we utilized the relationship between the smoothing parameter and “degrees-of-freedom” of Lindsay et al. [24] to tackle this problem algorithmically. Finally, after a full run of the Modal EM, each subset of observations that converges to the same mode are clustered together, and a modal representation of the landscape is obtained.

For convenience, we outline the steps of the clustering algorithm using a multivariate Gaussian kernel with covariance 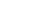 where 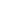 is the smoothing parameter. Let 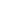 be the set of *D*-dimensional measurements based on chosen markers or scatters for a single cell in a flow sample. For notational simplicity we denote the kernel at 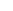 by 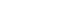 and present the following algorithm:

*MAC procedure in multiple dimensions*

Let the set of data to be clustered be 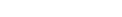 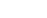 The clustering is performed as follows:

**Step 1.** Form kernel density.
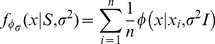
(1)using the (Gaussian) kernel 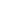


**Step 2.** Use 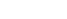 as the density function. Use each sample 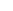 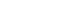 as the initial value in the Modal EM algorithm to find a mode of 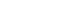 Let the mode identified by starting from 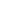 be 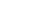


**Step 3.** Extract distinct values from the set 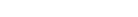 to form a set of modes *G*. Label the elements in *G* from 1 to 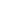 In practice, due to finite precision, two modes are regarded equal if the distance between them is below a certain threshold.

**Step 4.** If 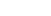 equals the 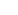 element in *G*, 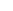 is put in the 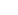 cluster.

As the clusters are formed by associating the observations with their corresponding modes, we call this procedure Mode Association Clustering (MAC). Any covariance structure for the kernel, be it Gaussian or otherwise, can be used to construct the modal cluster. The use of Gaussian kernel in our algorithm is motivated primarily by the the computational simplicity it provides (for details see Li et al. [20]). The resulting density not only maps the landscape but also provides soft clustering assignment to each observation. To be specific, if we denote the modal cluster *k*, 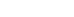 by 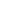 then the density estimation for cluster *k* is
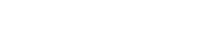



Without assuming any specific parametric form for the cluster densities, our MAC approach is more robust to unusual shapes and features (such as non-Gaussian tails) than than robust parametric clustering methods such as multivariate skew normal/t mixture models proposed recently by Lin [25] and Lin [26], and yet is based on reliable and fast EM estimation techniques.

### Enhancement of Modal Clustering: Modal Hierarchical Clustering

The notion of a “meaningful” population in a human expert’s understanding is often more complex than a simple isolated cluster of events. In flowScape, we address this complexity by enhancing the MAC procedure with a hierarchical framework to enable multiscale or multi-level resolution that we believe is better suited to emulate the nuanced human perspective. The hierarchical MAC procedure (called HMAC), and indeed any multiscale data analysis technique, presents an exciting new research area in statistics [27]–[30].

We note that when the bandwidth 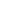 increases, the kernel density estimator 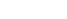 in (1) becomes smoother, and thus more points tend to climb to the same mode. This suggests a natural approach for hierarchical organization (or “nesting”) of our MAC clusters. Thus, given a sequence of bandwidths 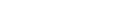 hierarchical clustering by HMAC can be performed in the following bottom-up manner.

Let the clustering of samples obtained at bandwidth level *l* be denoted as 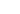 a function that maps sample 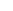 to the label of its cluster. Suppose *K* clusters labeled 1, 2, …, *K*, are formed at bandwidth 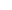. Then 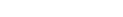. HMAC ensures that 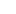
’s are nested, that is, if 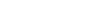 then 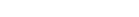 Denote the set of cluster representatives at level *l* by 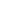 and its cardinality by 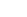 HMAC algorithm is described as follows:

*HMAC procedure in multiple dimensions*

**Step 1.** Start with the data 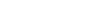 and set level 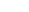 and initialize the mode association of the 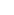 data point as 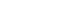


**Step 2.** 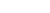.

**Step 3.** Form kernel density as in (1) using 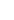.

**Step 4.** Cluster the points in 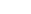 by using density 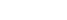. Let 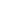 be the set of distinct modes obtained.

**Step 5.** If 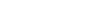 and the 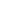 element in 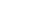 is clustered to the 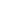 mode in 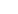 then 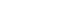 That is, the cluster of 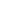 at level *l* is determined by its cluster representative in 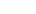


**Step 6.** Stop if 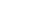 otherwise go to Step 2.

Importantly, while the number of objects being clustered reduces as we move up the hierarchy, the density estimator is always formed using all the original data samples, which has distinct advantages. Notably, HMAC differs from the traditional linkage-based hierarchical clustering, which also builds a hierarchy of clusters, in an important manner. In the linkage-based methods, only the two clusters with the minimum pairwise distance are merged, and the hierarchy is constructed as a sequence of such pairwise greedy merges, which are based on local comparisons. The lack of global analysis can result in skewed clusters (or “chain” sequences). In contrast, the merging of clusters in every level of HMAC is determined by a global criterion such that the contribution of every original data point on the overall clustering is retained through the density function 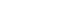


### Visualization of Flow Landscape: Use of Ridgelines

After preparing the above methodology for mapping a generic multi-dimensional data landscape, we adapted it for specific applications in flow data analysis. One such application is the use of *ridgelines*, an interesting mathematical device for facilitating the visualization of flow landscape especially in lower dimensions. As noted in Ray and Lindsay [31], ridgelines can be used for effectively connecting the modes and, importantly, to determine the connectedness or separation among the adjacent modal clusters.

In low-dimensions, flowScape uses ridgelines to provide an insightful representation of the overall landscape of flow data as fitted by the hierarchically structured modal clusters. Notably, by setting thresholds in the altitude (or “dip”) of a ridgeline at a particular level (or scale in the hierarchy), the user can separate and extract complex features easily and objectively. This, in fact, extends the human capacity since the user can now specify the level at which the population separation sufficiently matches her intuition. We can generalize this capability even further by allowing such thresholds to be user-specified “knobs”, thus flowScape can construct flexible templates to identify a collection of robust and suitable features in a semi-automated manner. By the nature of construction, such features can very effectively capture populations with unusual shapes or tails that may vary from sample to sample. Notably, they can be defined in relative terms, as opposed to only absolute population parameters (like physical location). The entire procedure can be regulated using visual feedback from density-based coloring at each point of the ridgelines. Interestingly, our ridgeline-based feature extraction procedure can be performed in high-dimensions.

In summary, modal clustering and its corresponding ridgeline analysis allow flowScape to exploit the geometry of a probability density function in a nontrivial manner. The steps of clustering can be conducted in accordance with our geometric heuristics, as described below. In particular, every modal cluster should be associated with a “hill”, and every point in a cluster can be moved to the corresponding hilltop along an ascending path without crossing the “valley” that separates two hills. Finally, by tracking the ridgeline between two peaks, the way in which two hills separate from each other can be measured and charted out, enabling diagnostics of our clustering results and also any adjustment of our clustering output as might be required for a particular flow data analysis.

#### Application of flowScape: Optimal Data Transformation

One of the key practical problems in flow data analysis, especially in the context of manual gating, is to ensure an optimal display of fluorescence intensities for different markers. Typically such marker distributions are log-normal, and thus log transformation is used for normalizing the data for visualization. While log_10_ transformation has been the norm in flow analysis, more recently other options have been considered for addressing several important issues on this topic [10], [32], [33]. Often a population of cells might have low mean or high variance due to practical reasons such as low expression of a specific marker, and hence these cells are essentially unstained or negative for that dye after correction for background fluorescence or spillover of fluorescence compensation. Yet the same cells could express properly for other markers and may be neither dead nor debris, and thus clearly represent valid events for further investigation. However, after compensation, they are distributed as negative clusters along those specific marker-dimensions, i.e. as a cluster with mean lower than 0, or with high variance such that the distribution extends well below 0.

Logarithmic transformation, however, is not defined on non-positive points, and therefore flow data displays quite often show a “log artifact” in which there is an artificial pile-up of points on the baseline. To address this, alternatives to log-scale displays, which nevertheless preserve many of the desired characteristics of log transformation, have been proposed. In general, a linear scaling is applied to the low end populations for spreading those events away from 0 at a rate faster than log-transformation. For points already farther away from 0, log-transformation is used. Such linear-log type transformations are usually symmetric around 0, applicable to negative values, and they smoothly transition from the faster linear spread to the gentler logarithmic for higher intensities (see Novo and Wood [32] and Trotter [33] for review of various transformations). Several transformations are implemented in the BioConductor package flowCore [9].

While transformations such as bi-exponential (e.g. logicle by Parks et al. [34]) or generalized inverse sine hyperbolic (Arcsinh) are quite useful for flow data, the task of optimizing the resulting display is finally based on the correct choice of the transformation parameters [10]. For instance, it is especially important is the determination of the correct value of the *cofactor* parameter which determines the spread of points away from the baseline where they may be piled up. Due to a sub-optimal value of cofactor, as we show below, a transformation may actually end up introducing spuriously split clusters of over-dispersed events. On the other hand, a cofactor could also be inadequate and the resulting transformation may fail to spread out the spikes in data. Either of these cases may lead to inaccurate analysis of the underlying populations. Importantly, the effect of a sub-optimal distribution along one marker-dimension can get propagated to higher dimensions, which can influence all steps of downstream multivariate analyses such as clustering and meta-clustering [1]. Therefore an optimal display must strike the right balance between both over- and under-transformation of compensated flow data.

To systematically address the problem of optimizing data transformation (ODT), we applied landscape mapping based on a new procedure flowScape.ODT. Unlike many flow analysis methods that rely on Gaussian densities and kernels for identifying populations, flowScape.ODT uses the more robust HMAC algorithm. There are two major advantages of this approach. First, untransformed data may not originally have Gaussian-like populations and thus may not conform to Gaussian models. Being free of the normality assumptions, flowScape.ODT can still identify these populations in the form of dense regions in the mapped landscape with precision. Further, it actually allows flowScape to utilize normality properties of the modified populations as statistical criteria for determining when a transformation has reached optimality. Indeed we combined multiple such criteria to test different aspects of what may be considered a “well-rounded cluster” such as unimodality, skewness and kurtosis. Clearly such determination would be either infeasible or redundant had we used Gaussian distributions in the first place for identifying the intermediate, not-yet-normal populations during the transformation process.

Our approach minimizes the redundancy in modifying the populations by observing that the rate of dispersion of points due to a log-like transformation gradually slows down away from 0. In other words, the choice of cofactor becomes increasingly less important for populations with high mean, i.e. the ones further away from the baseline. Hence, the criterion for an optimal transformation should primarily be concerned with any cluster that is located around 0 (besides the additional aim of removing the negative clusters, if any). Further, as noted in Parks et al. [34], since the display of compensated data can vary with the expression of marker-specific expression or dye-specific compensation, we apply flowScape to compute the optimal transformation parameter purposely on a per channel basis. That is, if an event needs corrective transformation for a specific marker, then our transformation does not needlessly alter that event’s proper expression for the other markers. In addition to making computation faster, the individual-channel approach of flowScape.ODT also allows us to select optimal values of cofactors within ranges that are distinctive to each marker or dye.

The flowScape.ODT procedure is based on the following steps:

- Based on the the sample’s landscape map for a given marker, flowScape identifies if there is any cluster with significant proportion (*p*) of points around the baseline 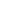 (“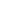-cluster” or “0-cluster” if 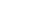) or less than 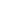 (“negative cluster”).
- The data are iteratively transformed with different values of the relevant parameter (such as increasing the Arcsinh cofactor) until there is no negative cluster – in other words, negative clusters are removed via transformation.
- The data are transformed with new values of the relevant parameter (e.g. cofactor) until the 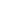-cluster is neither bimodal nor excessively peaked/flat/skewed – in other words, it should be unimodal and “rounded” enough to pass tests of normality such as Jarque-Bera test [35] and Hartigan’s Dip test [36].
- Once the optimal argument for the cofactor 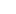 is determined, one can refine it further with a fast binary search using the scoring *S*(*T*) scheme (see Step 2) in the neighborhood of 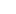, if desired.

Based on the above steps the algorithm is given by:

*flowScape.ODT – Optimal Data Transformation*

**Step 1.** For each channel or marker, we perform a transformation using an evenly-spaced sequence of cofactors 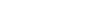 such that the start and end values (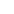 and 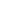) over- and under-transform the data by visual inspection.

**Step 2.** For each transformation 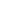 assign a score 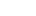 using the resulting clustering as follows:
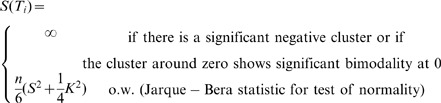
where 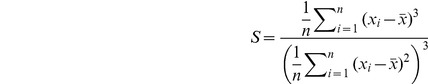 are the skewness and kurtosis of the cluster around 0.

**Step 3.** The optimal transformation is given by the cofactor.
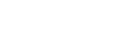



The above rules or guidelines can be easily fine-tuned according to one’s domain knowledge (for instance, the stains can influence one’s choice of cofactors) and understanding of the generated data (such as the effect of compensation for a specific dye). Thus, for instance, the baseline 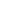 the proportion *p* and the boundary cofactors 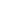 and 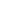 could be assigned in data-dependent manner. Likewise the significance (p-value) threshold for the normality criterion could be used to fine tune the optimal parameter selection.

#### Application of flowScape: Automated Gating based on Dynamic Templates

We now describe an algorithm that is suitable for automating manual gating. The map of the data landscape, as done by flowScape, can be a natural representation to capture the intuition behind manual gating since the populations could be viewed as dense regions of arbitrary shapes, sizes and locations spread over this landscape. For the purpose of applying our landscape-based approach in batch mode, we designed a new procedure flowScape.DTG. In the first step of the procedure, we construct a flexible template for one or more target populations in a sample. To support the flexibility, flowScape.DTG allows template specifications based on a mix of relative and absolute population characteristics. The templates are constructed by running our hierarchical modal clustering framework on some representative or training samples as supplied by the user. Subsequently, that learnt template is used to guide the identification and extraction of the corresponding target populations from a large batch of samples in a fully automated manner. Below we demonstrate the application by gating populations of live cel’ls and lymphocytes.

Our new procedure offers several novel features to tackle the problem of automated gating. First, we designed flowScape.DTG as a generic pattern-recognition procedure which can be used for extracting any subset of points – not just live cells or lymphocytes – that is identifiable in terms of either relative or absolute (or a mix of both) characteristics on the data landscape. Second, the unique advantage of flowScape lies in its use of hierarchical ordering of modal clusters, which allows it to isolate even complex populations with overlapping features that are otherwise much harder to demarcate automatically. The resulting template could thus be robust yet free of modeling constraints. Third, the templates could be specified in relative terms such as, “the 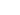 dense region along the positive *x*-direction”, somewhat similar to human intuition about a target population's location. Thus flowScape can effectively mimic a largely subjective operation with an objective procedure. Finally, flowScape.DTG leverages on the idea of a flexible template by applying it in a sample-specific, customizable form. The application of a given or learnt template to a batch of samples is dynamic in the sense that while its relative template characteristics are preserved, the corresponding physical instance could be revised according to the landscape map of every individual sample. Therefore, if “the 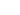 dense region along the positive *x*-direction” has a variable location, it is still gated accurately by flowScape.DTG. Indeed this helps our new procedure to tackle the well known problem of inter-sample variation in flow data in a systematic way.

For the live gating example, we constructed our temp’late based on the assumption that the live cell population is distributed in the 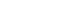 landscape further away from the origin (i.e. 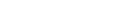) compared to the population of dead cells or debris. For our lymphocyte gating example, we constructed our template by assuming the second cluster away from the origin is our target population. It may be noted that variations of such rules for template construction can be specified easily to flowScape.DTG by the user based on either prior knowledge or in data-dependent manner. The semi-automated approach of flowScape.DTG's novel template construction offers considerable flexibility and robustness that are in general uniquely associated with manual gating. The algorithm is given by:

*flowScape.DTG – Dynamic Template based automated Gating*

**Step 1.** Find modes using HMAC in two dimensions, FSC and SSC, at multiple resolution 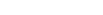 where 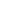
's are selected using the theory of pseudo degrees of freedom.

**Step 2.** Identify clusters with significant number of events based on a size threshold *s*. Let 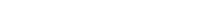 be the locations of cluster 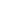 at the chosen level by the user.

**Step 3.** Calculate the distance 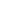 of cluster 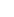 from the or’igin by 
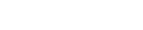
.

**Step 4.** For live gating identify the dead cell population as cluster 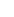 such that.
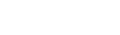



**Step 5.** For lymphgating, identify the lymphocyte population as cluster 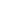 such that.
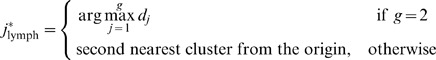



Although here we have described the application using only two dimensions (e.g. *FSC* and *SSC*), our algorithm works for any number of dimensions. Indeed a major advantage of flowScape’s generic description of clusters in the form of dense regions on a data landscape is that it allows us to characterize cell populations based on mixed types of observations. Unlike most flow analysis methods, the multi-dimensional landscape mapped by flowScape could be defined on a truly multivariate mix of scatters, stains, DNA content, etc. For instance, a template for live cells could be characterized not only with FSC-SSC but also with staining results from a viability assay. Similarly templates for different stages of cell proliferation or apoptosis could be defined by their DNA content along with, say, EdU staining from multiparametric immunofluorescence assays. Few other flow population identification algorithms offer such mix of flexibility and rigor.

### Description of Data

#### Regulatory T cell data (Treg)

The Treg data were originally generated and described in Maier et al. [37]. Here we used the samples that are available with the GenePattern package FLAME by Pyne et al. [1]. Peripheral blood mononuclear cells or PBMCs were stained with fluorophore-labeled antibodies against CD4, CD25, HLA DR, and Foxp3. Data were captured using a BD Biosciences FACSAria system. For preprocessing, a human operator performed live gating and logicle transformation.

**Table 1:** Optimal values of logicle cofactor for all markers in Treg Data

Variable	CD4	HLADR	CD25	Foxp3
Cofactor	3.5	3	3	3

#### Compensation Control data (CC)

Compensation control data were generated by staining a 1∶1 mixture of of positive (anti-Mouse Ig 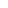) and negative (FBS) control compensation beads with mouse antibodies against human CD20 (clone H1) conjugated to PerCP-Cy5.5 and collecting approximately 4,000 events using a three-laser LSR II cytometer (Becton Dickinson). Data are publicly available on https://www.cytobank.org/cytobank/experiments/9748Cytobank. (https://www.cytobank.org/cytobank/experiments/9748).

#### Lymphoblastic Cell Line data (LCL)

The LCL data were originally generated and described in Choy et al. [38]. Lymphoblastoid cell lines (LCLs) were generated from unique individuals in the HapMap study, and stained with anti-HLA DQ and anti-CD95 antibodies. Data were captured with a BD Biosciences FACSCalibur system. Live gating and logicle transformation were performed with FLAME.

**Figure 1:**
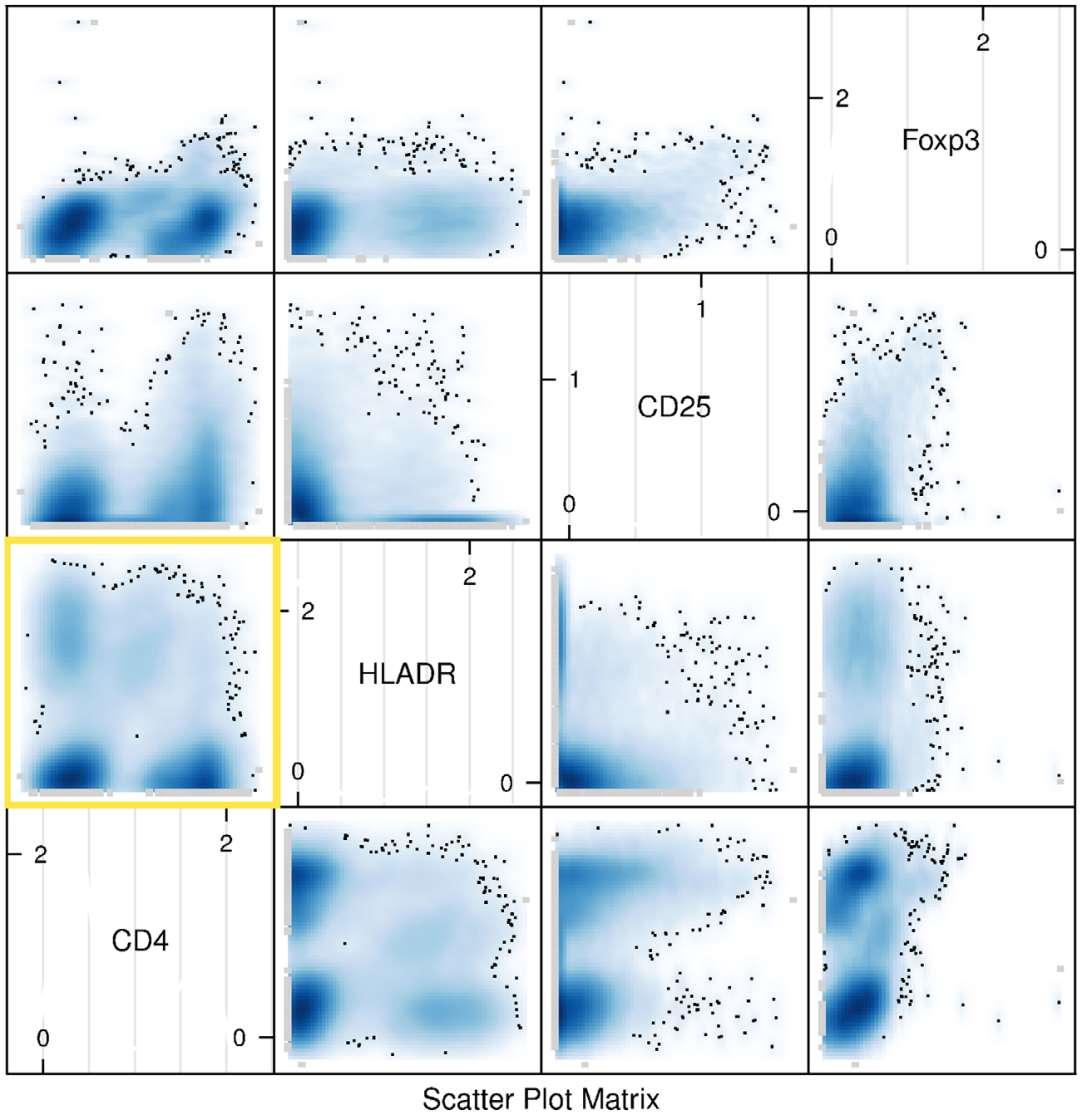
Optimal Transformation with flowScape. We show the distribution of Treg events after applying logicle transformation based on marker-specific optimal parameters computed with flowScape. The optimal arguments are shown in Table 1. Notably, for every marker, the distinct negative cluster has disappeared, and the existing clusters display approximately bell shaped distributions. All 0-clusters satisfy normality criteria for kurtosis, skewness and unimodality. The yellow box highlights the optimal structure consisting of fairly well-rounded populations.

#### Graft versus Host Disease data (GvHD)

The GvHD data were originally generated and described in Brinkman et al. [39]. Here we have used the subset available in the flowCore package of BioConductor and described in Hahne et al. [9].

**Figure 2:**
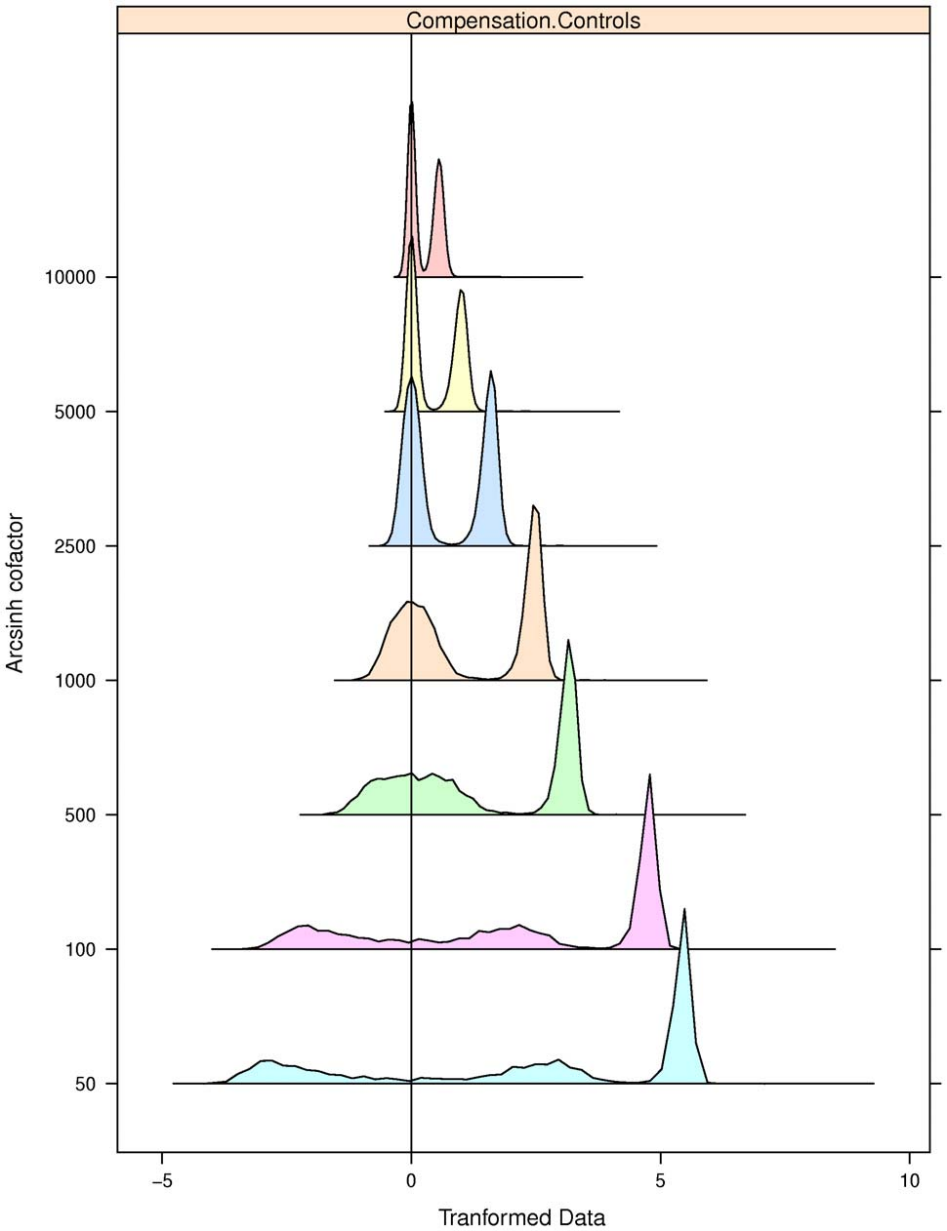
Selecting the optimal value of cofactor using flowScape. The distributions of CompControl events after Arcsinh transformation based on different values of the cofactor are shown. The cofactor values that satisfied our tests were 2500 and 1000. For these values, we see that there is no spurious splitting of the 0-cluster, which produces distinctive negative clusters for cofactors less than 1000. On the other hand, for cofactors greater than 2500, the 0-clusters are clearly spiky. In contrast, the 0-cluster for the cofactor values optimized according to flowScape normality criterion is neither too peaked nor too flat. Thus flowScape addressed both problems of over- and under-transformation of data.

## Results and Discussion

Flow cytometry is among the most popular in research and clinical labs around the world for several decades, yet only recently has computational cytomics started to receive major attention from the analytical scientists [40]. While a number of new algorithms have been developed recently for identification of cell populations in flow data, they generally lack a direct understanding or application of the human perspective of the manual gating process that they seek to automate. We understand that this is a difficult challenge for automated approaches, and a new robust approach may be necessary.

**Figure 3:**
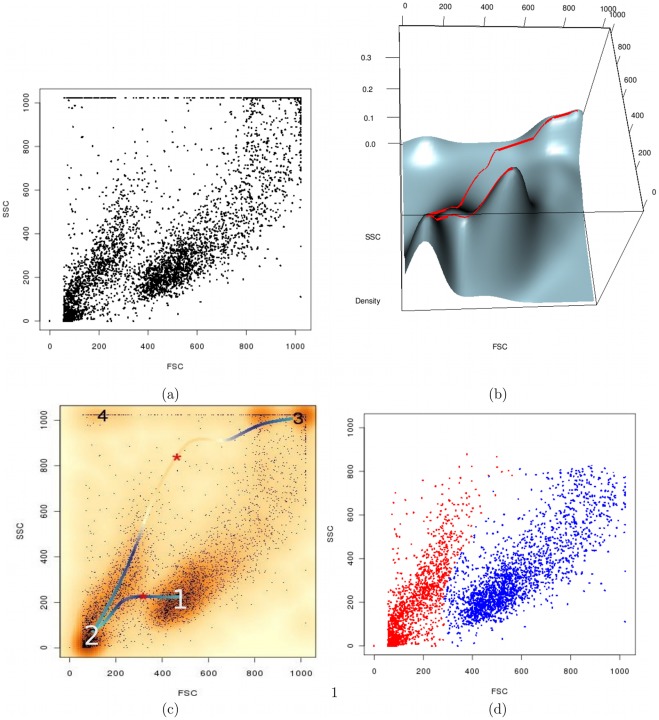
Modal clustering via landscape mapping and ridgeline analysis. We demonstrate live gating on a representative LCL sample using flowScape. (a) The sample is shown as a scatterplot in terms of forward and side scatters. (b) Using flowScape, we map the data landscape and determine the ridgeline (red curve) for the sample, as shown in 3-D. The ridgeline connects every modal cluster in the multi-dimensional data by traversing the terrain from peak to peak across slopes and valleys in terms of data density, thus providing a systematic hierarchical description of the sample using the landscape map. (c) The ridgeline (here shown as blue/yellow curve for dense/sparse regions) can therefore be used for objective extraction of relatively denser concentrations of events. A dip in the ridgeline (red asterisks) can guide the demarcation of cell subpopulations that are otherwise hard to isolate with automated clustering. Thus flowScape can offer the unique advantages of human intuition without paying the cost of associated subjectivity. (d) The final live gating results of flowScape are shown as 2 major populations in blue (live cells) and red (dead), after removing points at the extremity (around bin 1000). Clearly these clusters have non-elliptical shapes that could not be captured by many of the common clustering methods.

Through mapping of flow data landscape with hierarchical modal clustering and using algorithmic devices like ridgeline analysis and flexible templates, flowScape emulates the congregation-oriented view of data densities, which is free of pre-specified constraints on population shape. Based on the hierarchical representation, it also reflects the “zoom-in/zoom-out” approach of the human perspective. In future work, we want to create a semi-automated tool to implement the same approach with extensive interactive features.

**Figure 4:**
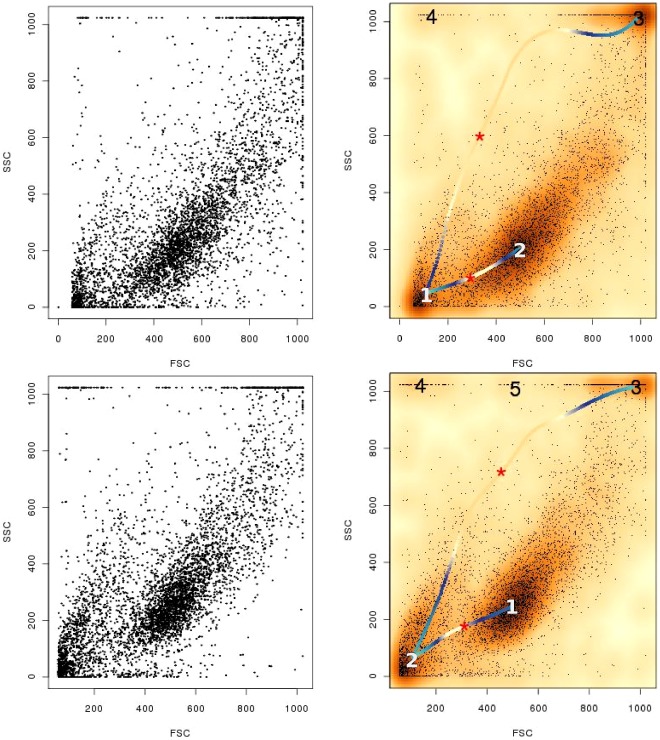
Objective isolation of cell populations. In the left panel we show the scatterplots of two LCL samples in terms of forward- and side-scatters. Owing to the inter-connected nature of the distributions, extraction of the live cell population is difficult via automation. Using modal clustering and ridgeline analysis, flowScape provides algorithmic means to separate and extract the populations based on locations where the altitude of the ridgeline dips while moving from one peak to another, as marked with red asterisks. The ridgeline is colored according to its altitude at each coordinate.

### Determining Optimal Transformation of Flow Data

In this section, we demonstrate the use of flowScape.ODT to determine the optimal transformations of two datasets: Treg and CC.

**Figure 5:**
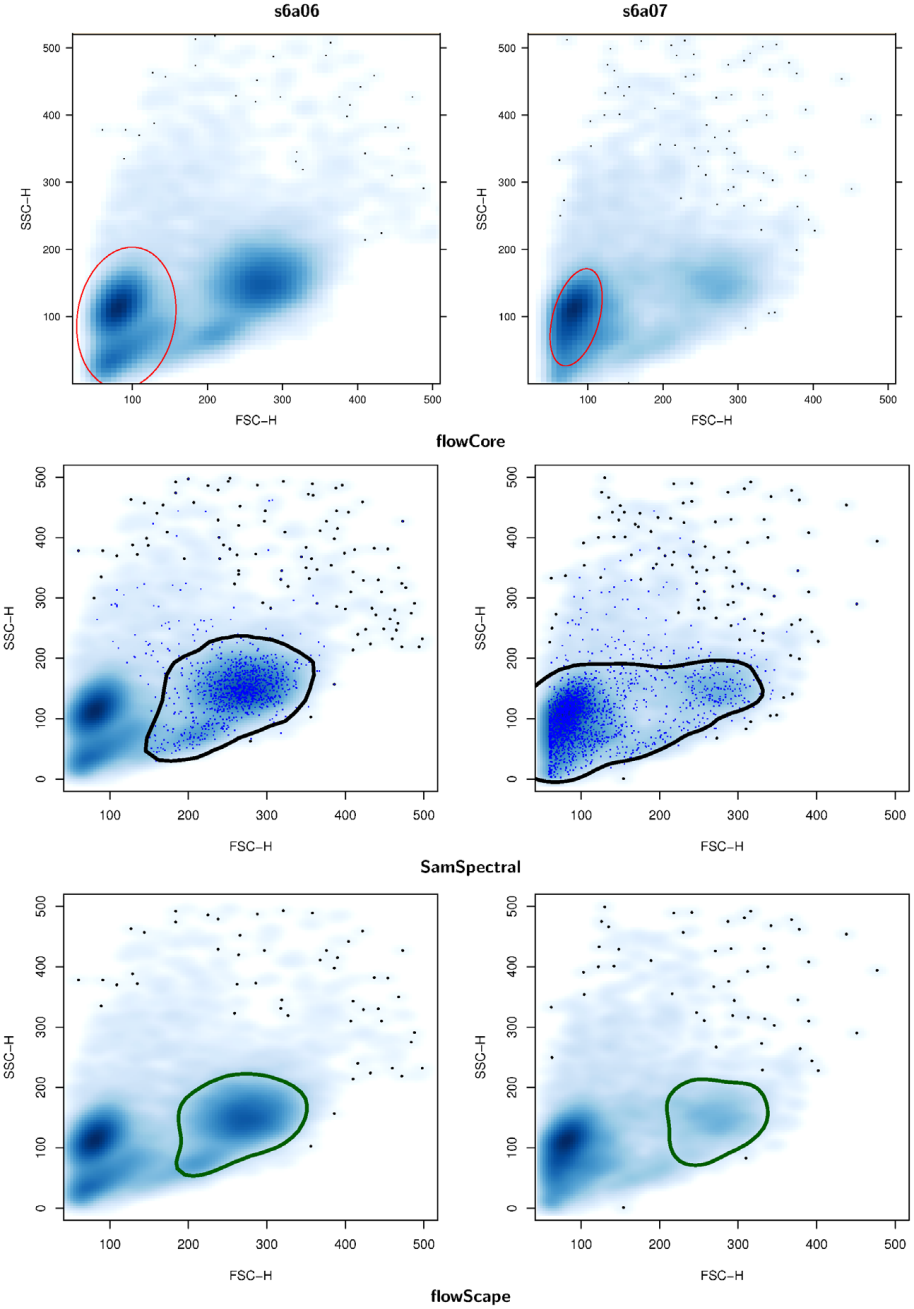
Comparative analysis of automated gating performance by different methods. We compared the results of lymphocyte gating for two representative samples (s6a06, s6a07 – the last two time points for Patient 6 in GvHD data). For both samples, we ran 2 well-known methods, flowCore and SamSpectral, and flowScape to automatically identify the lymphocyte populations (as defined in Ellis et al. [43]). While flowCore gate (red ellipse) was unable to detect the target population automatically in either sample, SamSpectral gated it (black outline) it correctly in only the sample to the left. On the other hand, due to the sparseness of the corresponding population in the sample to the right, SamSpectral failed to isolate it. In contrast, flowScape’s dynamic, sample-specific templates captured the lymphocyte populations accurately in both samples in spite of their inter-sample variation in locations, densities and shapes (green outline).

#### Transforming the Treg Data

When we applied the default logicle transformation to Treg data, for each of the markers Foxp3, CD25 and HLADR, we observed a significant “negative cluster” (Fig. S1). Then we tried to optimize the display with the flowTrans package, but the problem persisted (Fig. S2). To address the problem, we sought an optimal transformation that would remove the negative cluster while making the populations more normally distributed. Towards this we applied the flowScape.ODT procedure on Treg data to select the optimal cofactor for the logicle transformation over a range of values of this parameter independently for each marker (see Table 1 for results).

The distributions resulting from the optimally transformed Treg data is shown in Figure 1. For every marker, the distinct negative cluster has disappeared and the existing populations display densities that are closer to a bell shape. In contrast to the sub-optimal display of other methods, our optimal structure in form of fairly well-rounded non-negative clusters is highlighted in Figure 1 (see yellow box). All the 0-clusters satisfy the Dip Test for unimodality and Jarque-Berra test for normality based on skewness and kurtosis. Clearly compared to the transformation obtained using optimal value selected by the FlowTrans package (see Figure S2 in Supplementary Materials for flowTrans output), our method does a superior job in both removing the negative cluster as well as making the 0-clusters more normal-like. We found, in fact, the optimized output from FlowTrans is probably not too different from the logicle-transformed output with default untransformed parameters. Both contain negative clusters that are hard to interpret undermining the very purpose of a transformation. Notably, FlowTrans provides only a common optimal parameter value for all the different markers although it is well-known that some dyes and/or markers (like CD4 in the present case) may actually need different extents of adjustment depending on the particular run on a given cytometer. The output from flowScape addresses this issue by considering the various arbitrary-shaped clusters that appear in the intermediate stages of transformation using different parameter-values. Being free of parametric specifications, flowScape can utilize normality of the resulting distributions (the zero-populations, in particular) as both an intuitive as well as rigorously testable criterion.

#### Analysis of Compensation Control Data

Next we applied the flowScape.ODT procedure to the CC dataset. Here we have just one variable corresponding to two artificial populations of a 1∶1 mixture of positive and negative compensation control beads stained with PerCP-Cy5.5. The cytometer settings placed the center of the distribution near zero, making this an excellent example for issues with events near and below 0. The data were transformed according to Arcsinh (i.e. inverse sine hyperbolic) function over a range of values of cofactor *c* [41]. The function is defined as: Arcsinh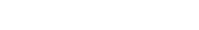. The Arcsinh-transformed distributions for the CC data are shown in Figure 2. While increasing the cofactor arguments in a sequence of values from 50 to10000, we found that two values, 1000 and 2500, satisfy the tests of normality. For cofactor of 1000, the transformed data contained a “gently-rounded” 0-cluster, whereas for 2500, the 1∶1 mixture of populations was apparent. There was no distinct negative cluster in either case. If the user is interested, she could opt for running flowScape.ODT with successively finer ranges of cofactor arguments, such as zooming in between 1000 and 2500. The optimality is clearly evident from the resulting display which avoids the spurious splitting of the 0-cluster as caused by larger choices of cofactor values (Figure 2). Thus, flowScape.ODT solved the dual problems of over- and under-transformation of flow data.

### Application of Livegating to LCL Data

We applied the automated gating procedure flowScape.DTG for live gating of LCL data. Here we demonstrate the results using a representative sample. (The full set of results are available from the authors upon request.) The data dimensions that we used for live gating are forward and side scatters. The 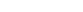 scatterplot is shown in the Figure 3(a) and the final results of flowScape.DTG gated live (versus non-live) population is shown in Figure 3(d). While the final results may appear as cleanly separated into live and non-live clusters, the complex, non-spherical shapes of the populations identified by flowScape.DTG are proof of our robust hierarchical representation and systematic ridgeline analysis as described in the Methods.

First, we mapped the 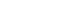 data landscape to detect the relatively dense regions as modal clusters connected with a ridgeline (see Figure 3(b)). Using the ridgeline to guide the elevation of peaks and valleys in the landscape, we identified the individual clusters (numbered 1 to 4 in Figure 3(c)) specific to this particular sample. We color-coded the elevation along the ridgeline (blue/yellow for high/low elevation) in Figure 3(c) and also marked the points of minima along the curve with red stars. An insightful representation of our landscape mapping and ridgeline tracing approach is available through a 3-dimensional view that offers a clearer perspective (Figure 3(b)) in terms of relative densities of the major and minor populations. While we understand that further validation is necessary to confirm live cells, such as with viability markers, our primary goal here is to present objective means to complement the human subjectivity used for identifying populations of different shapes and structure. Indeed given the complementary aims, we chose not to introduce any bias by using manual gating for benchmarking our results. More examples of live-gating are given in Figure 4.

As noted, unlike other live gating approaches [42], the dead and the live cell clusters obtained by our approach are not Gaussian in shape. In fact these are even more flexible than densities given by classes of elliptical or skewed elliptical distributions. This property makes our approach ideally suited for direct, automatic extraction of previously unidentifiable, complex cluster shapes. First, to emulate the “top down” perspective, we identify concentrations of events around different modes as modal clusters. Second, in a “bottom up” fashion, these clusters can be connected via a hierarchical representation to model complex structures. To enable that, our ridgeline analysis provides a valuable objective mechanism to determine the separation of two or more populations. As future work, we plan to create a semi-automated tool that will allow the flowScape user to perform concrete yet rigorous operations with the clusters, the ridgeline and the different parameters to construct complex gating templates within a highly interactive format.

### Application of Lymphocyte Gating to GvHD Data

We applied flowScape.DTG’s dynamic, sample-customized gating methodology to GvHD data. In principle, the clustering and the ridgeline analysis steps of lymphgating are similar to livegating except for the different definitions of the dynamic template in each case. For instance, for lymphgating, we defined the lymphocyte template as the population whose mode is the second farthest from the origin in terms of Euclidean distance in 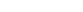 event space. This takes into account the low size (*FSC*) and granularity (*SSC*) of the dead cells and debris that would appear close to the left extremity of the scatterplot. We note that this definition is relative, and not specified by any absolute co-ordinates or boundaries. Thus both the location and the shape of the target lymphocyte population to be detected by flowScape.DTG can be flexible.

The flexibility of the flowScape.DTG templates allows highly robust automated detection of cell populations, even in the presence of platform noise, high-inter-sample variation, sparse or diffuse populations, etc. To illustrate this point, we selected two consecutive time-points measured in the same patient from GvHD dataset (s6a04,s6a05), and applied flowScape.DTG as well as other methods (Figure 5). Our objective was to automatically detect the lymphocyte population, which are typically characterized by their higher size and granularity as compared to dead cells that are closest to the origin. These samples serve as good examples of how despite being two consecutive time-points measured in the same patient, one of them (the left sample) has a prominent lymphocyte population whereas the same is very sparse in the other (the right sample). The results of automated detection are shown in Figure 5. First, we see that the lymphgate function in the flowCore package [9], [43], which uses a Gaussian kernel (red outlines), clearly missed the target population in both samples. While the detection (shown with black outlines) improved with the SamSpectral method [44] for the left sample, it failed for the right sample owing to the sparseness of the target population. Using the robust, sample-specific application of templates, flowScape.DTG could however detect both the prominent population in the left sample as well as the rare one in the right sample (green outlines). Notably, our template need not be rigidly elliptical or even symmetric in shape, although if the population does have such a shape, then flowScape.DTG will closely approximate it. Again, whether the sparse cluster in the right sample truly represents lymphocytes cannot be validated solely by computational analysis of 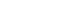 scatterplot, but our point is to highlight the robustness of flexible templates in detecting populations even if they are noisy, sparse, or of variable form and location (for another example with two subclusters of the same lymphocyte population see Figure S3 in Supplementary Materials).

### Conclusions

Understanding the human perspective in thinking about and making sense of visual information, as in the steps of manual gating, is a complex problem. When a flow cytometry analyst visualizes the data, a complex interplay between human intuition and technical understanding (both biological and mechanical) is brought into action. While such insight may be difficult, if not impossible, to reproduce outside the human mind, we can try to emulate certain aspects of it via automation. For instance, the zooming in/out approach could be captured with a data representation that has multi-level resolution. Toward this, we used flowScape to utilize the notion of a modal cluster to offer a congregation-oriented view of the data landscape. The resulting map of the data landscape uniquely emulates the global overview of a human analyst but it does so with a mathematically rigorous density function. Then we use a bottom-up hierarchical representation of the modal clusters to mimic the manual construction of complex structures at multi-level resolution. Thus we try to capture certain amount of the subjectivity of the human perspective, and the strength it brings to manual flow data analysis, via our objective means. Finally, we extended the manual gating capacity with our novel flexible, sample-specific templates for extracting features of interest which may have unusual shapes and distributions and are possibly difficult to isolate using other computational methods.

## Supporting Information

Figure S1 **Results of application of logicle transformation with default arguments.** We plot the distribution of Treg events after applying logicle transformation based on its default parameter values, i.e. without any transformation. We note that the resulting transformation did not remove the negative cluster (left of 0) in any of the four markers. Apparently there is little difference between these results and the ones due to logicle transformation with flowTrans-optimized argument in Figure S2.
(TIF)

Figure S2 **Results of transformation with the flowTrans package.** We plot the distribution of Treg events after applying logicle transformation based on a single parameter that was optimized according to the flowTrans package. We note that the resulting transformation did not remove the negative cluster (left of 0) in any of the four markers. Apparently there is little difference between these results and the ones due to logicle transformation with default (non-optimized) arguments in Figure S1.
(TIF)

Figure S3 **flowScape gating in the presence of multimodal lymphocyte cluster.** We present the results of lymphocyte gating for a representative sample (s7a06 – last time points for Patient 7 in the GvHD data) to demonstrate how flowScape allows us to merge two subclusters of the same lymphocyte population using the flowScape algorithm. Among these two samples. The flowScape gating is given by the bold green line whereas the subclusters are marked by the density contour plots (dotted black) of the two subclusters. Here the the two adjacent modes given by the contour were combined in the cluster hierarchy to create the lymphocyte cluster given by the solid green line.
(TIF)
