# Supplementary figures and images for: A Computational Framework to Emulate the Human Perspective in Flow Cytometric Data Analysis
Source: PLoS One. 2012 May 1;7(5):e35693. doi: 10.1371/journal.pone.0035693 (PMC3341382; doi:10.1371/journal.pone.0035693)

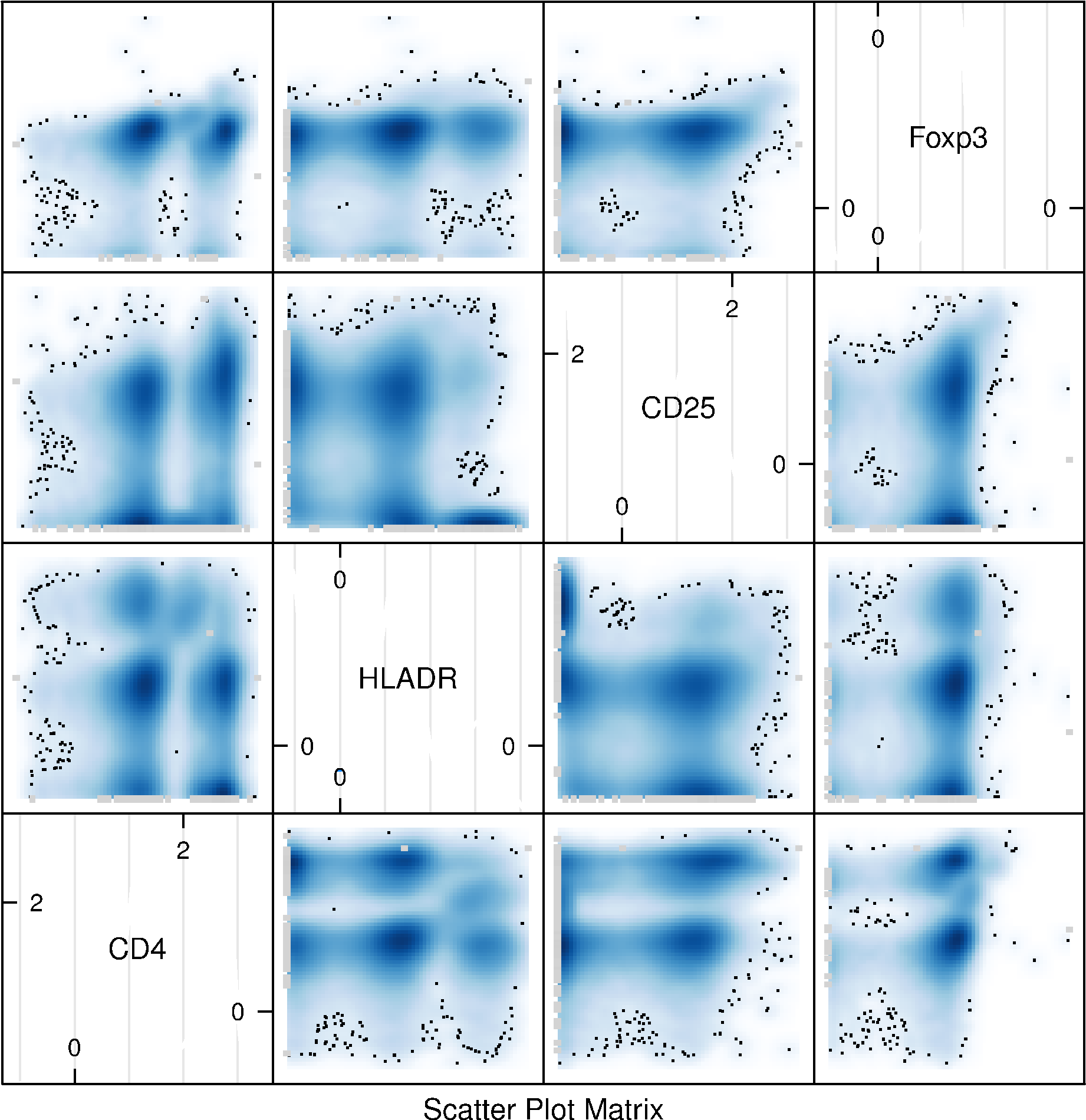

Supplement: Figure S1 — Results of application of logicle transformation with default arguments. We plot the distribution of Treg events after applying logicle transformation based on its default parameter values, i.e. without any transformation. We note that the resulting transformation did not remove the negative cluster (left of 0) in any of the four markers. Apparently there is little difference between these results and the ones due to logicle transformation with flowTrans-optimized argument in Figure S2. (TIF) [file pone.0035693.s001.tif]

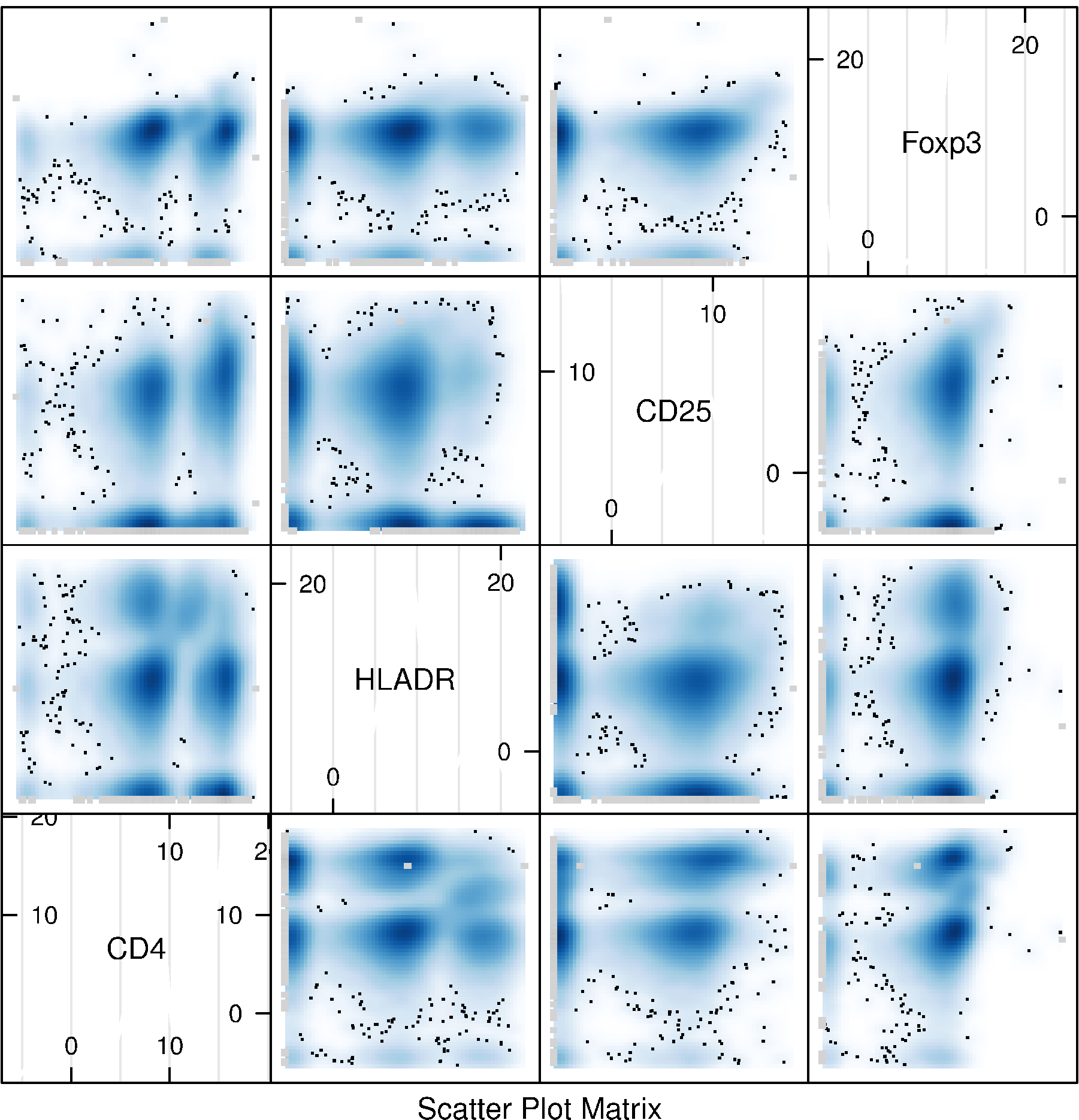

Supplement: Figure S2 — Results of transformation with the flowTrans package. We plot the distribution of Treg events after applying logicle transformation based on a single parameter that was optimized according to the flowTrans package. We note that the resulting transformation did not remove the negative cluster (left of 0) in any of the four markers. Apparently there is little difference between these results and the ones due to logicle transformation with default (non-optimized) arguments in Figure S1. (TIF) [file pone.0035693.s002.tif]

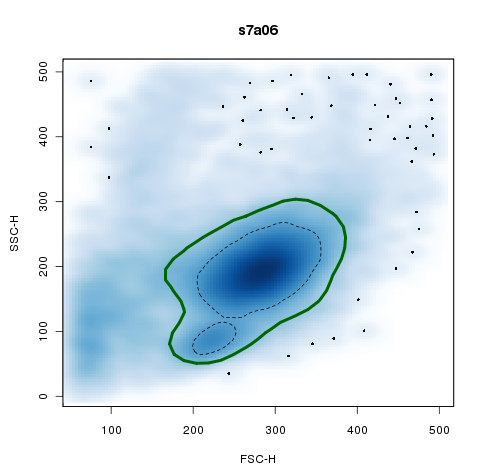

Supplement: Figure S3 — flowScape gating in the presence of multimodal lymphocyte cluster. We present the results of lymphocyte gating for a representative sample (s7a06 – last time points for Patient 7 in the GvHD data) to demonstrate how flowScape allows us to merge two subclusters of the same lymphocyte population using the flowScape algorithm. Among these two samples. The flowScape gating is given by the bold green line whereas the subclusters are marked by the density contour plots (dotted black) of the two subclusters. Here the the two adjacent modes given by the contour were combined in the cluster hierarchy to create the lymphocyte cluster given by the solid green line. (TIF) [file pone.0035693.s003.tif]
